# Dose-Dependent Effects of Nickel on Skeletal Development: Physiological Necessity and the Threshold of Toxicity

**DOI:** 10.3390/ijms27104538

**Published:** 2026-05-18

**Authors:** Xiaoxin Ma, Xi Huang, Jinyu Li, Lixian Wu, Runxin Zhang, Daqi Huang, Li Gao, Chuanjiang Zhao

**Affiliations:** Hospital of Stomatology, Sun Yat-sen University, Guangzhou 510055, China

**Keywords:** nickel, dose, integrin-linked kinase, AKT–mTOR signaling, bone development

## Abstract

Nickel (Ni) is a ubiquitous trace metal, yet its physiological dynamics and dose-dependent roles in skeletal biology remain unclear. Here we combined elemental mapping, cellular assays, multi-omics and mouse models to define how Ni availability modulates osteogenesis. Ni, together with Manganese (Mn), chromium (Cr) and copper (Cu), was readily detectable in serum from both mice and humans. In situ LA–ICP–MS further showed that Ni levels in embryonic calvaria rose significantly across stages and CaO exhibited a consistent upward trend, suggesting coordinated accumulation of Ni with cranial mineralization. In vitro, Ni exerted biphasic effects on bone marrow mesenchymal stromal cells (BMSCs): high-dose Ni (100 μM) suppressed proliferation, elevated ROS, and induced time-dependent upregulation of *Hmox1* and *Nos2*, consistent with escalating oxidative/nitrosative stress. By contrast, low-dose Ni (0.1 μM) enhanced matrix mineralization, whereas this pro-mineralization effect was attenuated at higher concentrations. In vivo, both Ni deprivation and Ni overload impaired bone formation: a Ni-free diet caused trabecular rarefaction and reduced mineral apposition, while high Ni hindered bone development of mice, especially in the early-stage intake. Mechanistically, RNA-seq and Ni-NTA proteomics identified Ni-driven osteogenic transcriptional remodeling and increased Ni-binding proteins, prioritizing integrin-linked kinase (ILK) as a Ni-inducible binder. ILK was required for osteogenic differentiation, and low-dose Ni activated AKT–mTOR signaling in an ILK-dependent manner. Finally, low-dose Ni-pretreated collagen scaffolds enhanced calvarial defect repair. Together, these findings define a narrow physiological window in which Ni supports osteogenesis via ILK–AKT–mTOR, whereas both deficiency and excess disrupt skeletal accrual.

## 1. Introduction

Bone is a dynamic organ that provides mechanical support and serves as the primary reservoir for mineral homeostasis. Its development and regeneration depend on tightly coordinated cellular events, encompassing osteoprogenitor proliferation, osteogenic differentiation, extracellular matrix deposition, and mineralization [1,2]. Beyond the canonical roles of calcium (Ca) and phosphorus (P), accumulating evidence underscores that essential trace metals, including zinc (Zn), Cu, and magnesium (Mg), are integral to skeletal biology [3]. Acting as enzyme cofactors and signal modulators, these micronutrients govern matrix synthesis and maturation, dictating bone formation and structural integrity. Elucidating how trace metals regulate osteogenesis is thus pivotal for understanding physiological skeletal development and pathological bone disorders.

Nickel (Ni) is a ubiquitous environmental metal extensively utilized in industrial applications owing to its favorable physicochemical properties [4]. Consequently, trace levels of Ni are routinely detectable in human biofluids and tissues, including blood, urine, and multiple organs [5,6,7]. At elevated exposure levels, Ni is notorious for deleterious health effects, spanning contact dermatitis and pulmonary fibrosis to adverse developmental outcomes [4]. Intriguingly, whether Ni qualifies as a physiologically relevant trace element in humans remains fiercely debated [8,9]. Although serum Ni concentrations below ~2 ng/mL constitute a proposed physiological baseline [10], mechanistic insights delineating the cellular targets and molecular pathways of Ni at these trace levels remain strikingly scarce. Existing studies are disproportionately skewed toward the toxic effects of nickel, mainly focusing on oxidative stress, allergic responses and carcinogenicity induced by high-dose nickel exposure [4,11,12,13,14,15], leaving its potential biological functions at physiological concentrations largely unresolved.

In skeletal tissues, trace elements such as Zn, Mg [16,17], and selenium (Se) [18] act as critical regulators of bone metabolism by modulating osteogenic transcription factors (e.g., *Runx2*, *Osterix*) and key signaling cascades (e.g., BMP/Smad, Wnt/β-catenin) [3,19]. By contrast, despite its pervasive in vivo presence, the contribution of Ni to bone development and regeneration, along with the underlying molecular mechanisms, remains poorly defined.

To address this critical knowledge gap, we quantified trace metals in murine and human serum, confirming their consistent presence under physiological conditions. By leveraging an established mouse calvarial development model coupled with in situ inductively coupled plasma mass spectrometry (ICP-MS), we demonstrated that Ni content in embryonic calvaria increased progressively from E12.5 to E15.5, exhibiting a robust consistent trend with calcium deposition. These findings indicate that Ni accumulation actively accompanies, and potentially drives, normal skeletal development. Thus, we hypothesize that Ni exerts a biphasic, dose-dependent influence on bone homeostasis: physiological Ni concentrations promote osteogenesis and facilitate bone regeneration; Ni deprivation impairs skeletal development and reduces bone mass; and excessive Ni exposure disrupts osteoblast function, compromising overall skeletal integrity.

## 2. Results

### 2.1. Trace Metal Levels in Serum and Developmental Accumulation of Ni in Embryonic Calvaria

**Figure 1:**
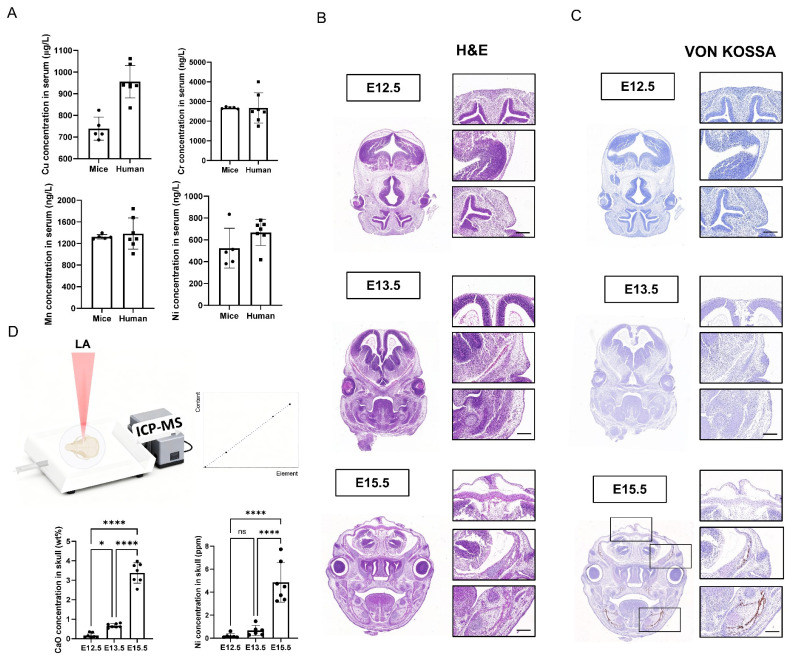
Trace metal levels in serum and developmental accumulation of Ni in embryonic calvaria. (**A**) Quantification of Ni, Mn, Cr, and Cu in serum from mice and humans. (**B**) Representative H&E staining of mouse head sections at E12.5, E13.5, and E15.5. Scale bar = 200 μm. (**C**) Von Kossa staining of adjacent sections showing progressive mineral deposition/ossification. Scale bar = 200 μm. (**D**) Schematic of LA-ICP-MS and quantification of CaO and Ni in embryonic skulls. Statistical significance was indicated as shown in the figure. * *p* < 0.05, **** *p* < 0.0001, ns = not significant.

To define the physiological presence of trace metals, we quantified Ni, Mn, Cr and Cu in serum from mice and humans. All four metals were readily detectable in both species (Figure 1A). To visualize craniofacial development across embryogenesis, hematoxylin and eosin (H&E) staining was performed on coronal sections of mouse heads at E12.5, E13.5, and E15.5, revealing progressive maturation of cranial tissues and the developing calvarial structures (Figure 1B). In parallel, Von Kossa staining demonstrated a stage-dependent increase in mineral deposition within the cranial region, consistent with ongoing ossification and bone matrix mineralization during this developmental window (Figure 1C). We next applied in situ laser ablation inductively coupled plasma mass spectrometry (LA-ICP-MS) to quantify elemental content in embryonic calvarial tissue. Notably, Ni levels in the skull increased significantly from E12.5 to E15.5, mirroring the rise in mineral-associated calcium (reported as CaO) (Figure 1D).

### 2.2. Nickel Deprivation and Overload Both Suppress Osteogenic Mineralization in a Stage-Dependent Manner

**Figure 2:**
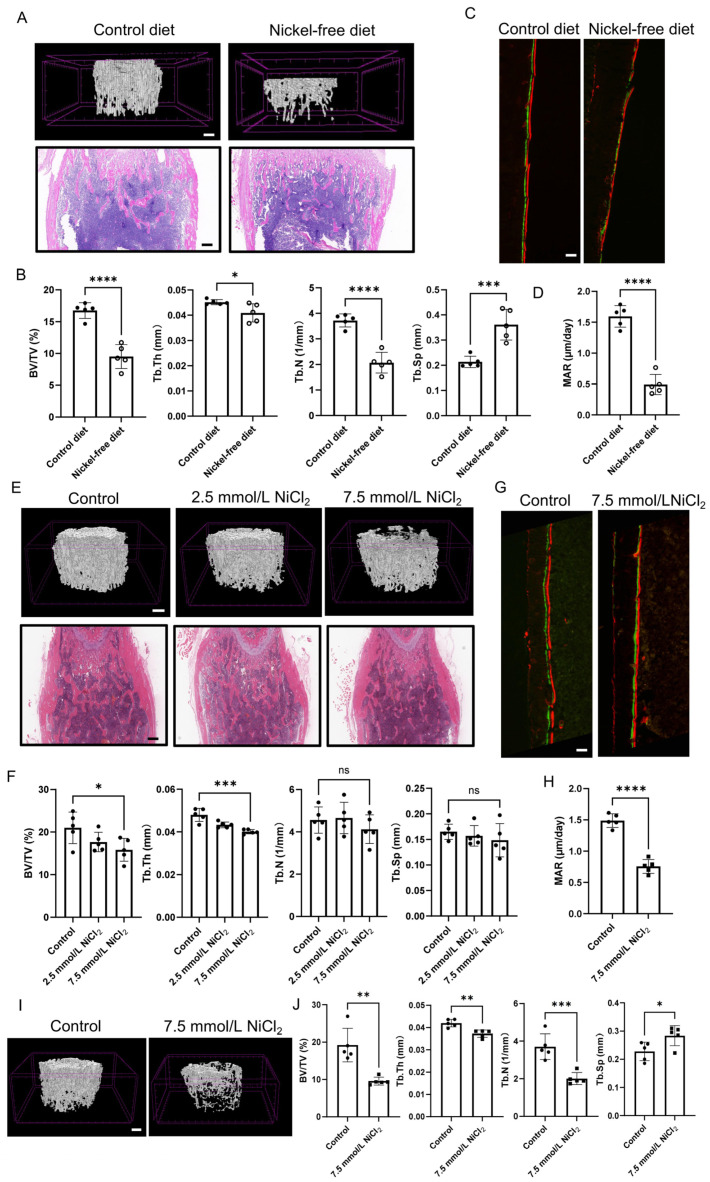
Nickel-free diet and high nickel exposure impair femoral trabecular architecture and reduce mineral apposition in mice. (**A**) Representative femoral trabecular micro-CT 3D reconstructions and H&E-stained sections from control diet-fed mice and nickel-free diet-fed mice, showing trabecular rarefaction in the nickel-free group. Scale bar = 250 μm. (**B**) Micro-CT analysis of trabecular morphometric parameters (BV/TV, Tb.N, Tb.Th, Tb.Sp) comparing control versus nickel-free diet groups. (**C**) Representative bone histomorphometry images following double fluorochrome labeling (calcein, green; alizarin red, red). Scale bar = 100 μm. (**D**) Quantification of mineral apposition rate (MAR) between femurs from nickel-free diet-fed mice and controls. (**E**) Representative images of 3D construction and H&E staining of femurs from mice receiving control water, low-nickel water (2.5 mmol/L), or high-nickel water (7.5 mmol/L). Scale bar = 250 μm. (**F**) Micro-CT quantification of femurs in the high-nickel (7.5 mmol/L) group compared with control in the late stage of nickel intake (From the fifth week after birth). (**G**) Representative calcein/alizarin labeling images showing decreased inter-label distance in mice exposed to high-nickel water (7.5 mmol/L). Scale bar = 100 μm. (**H**) Quantification of MAR between femurs from the high-nickel (7.5 mmol/L) group and controls in the late stage of nickel intake (From the fifth week after birth). (**I**) Micro-CT quantification of femurs in the high-nickel (7.5 mmol/L) group compared with control in the early stage of nickel intake (From the second week after birth). Scale bar = 250 μm. (**J**) Quantification of MAR between femurs from the high-nickel (7.5 mmol/L) group and controls in the early stage of nickel intake (From the second week after birth). * *p* < 0.05, ** *p* < 0.01, *** *p* < 0.001, **** *p* < 0.0001.

Mice fed a nickel-free diet exhibited a markedly smaller body size relative to those on the control diet (Figure S1A,B). To determine whether dietary nickel is essential for bone development, femurs from mice fed a nickel-free diet were analyzed via micro-computed tomography (micro-CT) and H&E staining. Results revealed a marked trabecular rarefaction in nickel-free diet-fed mice relative to controls (Figure 2A). Consistent with these findings, micro-CT morphometric analyses demonstrated significant reductions in bone volume fraction (BV/TV), trabecular number (Tb.N), and trabecular thickness (Tb.Th), alongside a concomitant increase in trabecular separation (Tb.Sp) in the nickel-free diet group (Figure 2B). To directly interrogate bone formation dynamics, calcein was intraperitoneally injected into mice, followed by alizarin red injection five days later. Nickel-free diet-fed mice exhibited a markedly reduced distance between the two fluorochrome labels (Figure 2C,D), indicating that nickel deprivation suppresses osteogenic mineralization in vivo.

We next investigated whether nickel excess perturbs bone formation and whether this effect is dependent on the developmental stage of exposure. In the late-stage nickel intake model, femurs from the 7.5 mmol/L NiCl_2_ group appeared smallest (Figure S1C). Micro-CT and H&E staining showed a visibly diminished trabecular network in the high-nickel group relative to control mice (Figure 2E). Quantitative analysis revealed significantly lower BV/TV and Tb.Th in the high-nickel group versus the control group (Figure 2F), and calcein/alizarin red double-labeling demonstrated a significant reduction in the mineral apposition rate (MAR) (Figure 2G,H).

To determine if bone is more susceptible to nickel toxicity during earlier developmental windows, we analyzed mice with early-stage nickel intake. Micro-CT imaging revealed that early exposure to 7.5 mmol/L NiCl_2_ induced severe trabecular bone loss (Figure 2I). Morphometric analysis confirmed drastic reductions in BV/TV, Tb.Th, and Tb.N, with a significant increase in Tb.Sp compared to controls (Figure 2J). Intriguingly, comparative observation indicated that the adverse effect of high-concentration nickel on bone mass was far more pronounced in the early-stage intake group than in the late-stage group, suggesting a higher susceptibility of developing bone to nickel overload. Furthermore, immunofluorescence analysis of femoral sections revealed that mice receiving high-concentration nickel exhibited a marked reduction in RUNX2, ALP and COL1A1 fluorescence intensity compared with control mice (Figure S2A–C). Together, these results support a dose- and stage-sensitive effect of nickel on skeletal biology: nickel deprivation compromises physiological trabecular accrual, whereas nickel excess imposes a strong osteotoxic effect that suppresses mineral apposition, with early-stage exposure causing more severe skeletal deficits.

### 2.3. Nickel Exerts Dose-Dependent Effects on BMSC Proliferation and Osteogenic Mineralization

**Figure 3:**
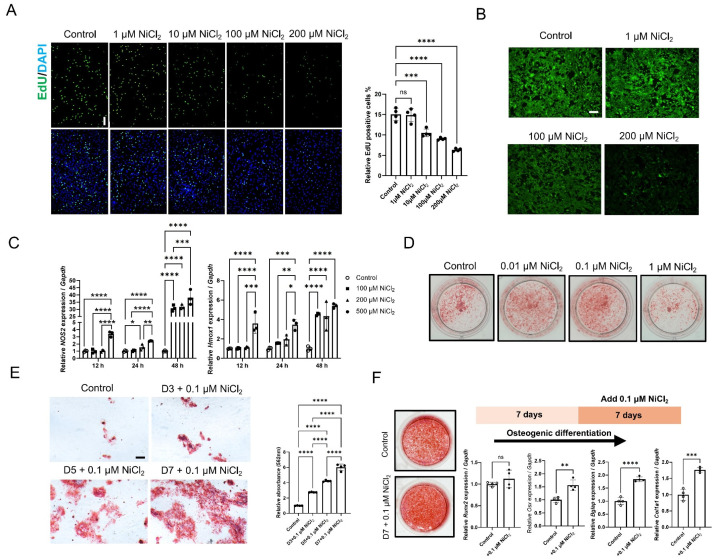
Dose-dependent effects of nickel on BMSC proliferation, nickel uptake, stress-response gene expression, and osteogenic mineralization. (**A**) EdU incorporation assay in BMSCs treated with nickel (1 μM, 10 μM, 100 μM and 200 μM). Representative fluorescence images show EdU (green) and nuclei counterstained with DAPI (blue). (**B**) Calcein-based tracking of intracellular nickel accumulation. (**C**) qPCR analysis of stress-response genes (*Hmox1* and *Nos2*) in BMSCs stimulated with nickel at the indicated concentrations and durations. (**D**) Alizarin Red S staining of BMSCs in osteogenic conditions with different concentrations of nickel including 0.01 μM, 0.1 μM and 1 μM. Scale bar = 200 μm. (**E**) Representative Alizarin Red S staining images showing calcium mineral deposition after 14 days of osteogenic induction. Scale bar = 200 μm. Cells were treated with 1 μM Ni during the different days. (**F**) RT–qPCR analysis of BMSCs in osteogenic conditions during the late phase (Day 7–14) of 0.1 μM Ni exposure. * *p* < 0.05, ** *p* < 0.01, *** *p* < 0.001, **** *p* < 0.0001.

EdU incorporation assays demonstrated that exposure to 200 μM Ni significantly suppressed cell proliferation, whereas 1 μM Ni showed no detectable adverse effect compared with controls (Figure 3A). Calcein fluorescence quenching confirmed substantial intracellular Ni accumulation at 100 and 200 μM, validating the capacity of BMSCs to internalize Ni under high-burden conditions (Figure 3B). Regarding oxidative stress, intracellular ROS in BMSCs was markedly increased by 100 μM NiCl_2_ after 24 h (Figure S3). Time-course qPCR analysis revealed that high-concentration Ni triggered a progressive induction of *Hmox1* and *Nos2*, indicative of escalating oxidative/nitrosative stress (Figure 3C). Consistent with these in vitro findings, in vivo experiments confirmed that high-concentration Ni intake significantly upregulated HO-1 (gene: *Hmox1)* and iNOS (gene: *Nos2)* expression in murine femoral tissues (Figure S4A,B), mirroring the stress response observed in BMSCs.

In terms of osteogenic capacity, Alizarin Red S staining revealed a biphasic dose response: 0.1 μM Ni enhanced extracellular matrix mineralization, whereas this pro-mineralization effect was attenuated at higher concentrations (e.g., 1 μM) (Figure 3D). Furthermore, late-phase addition of 1 μM Ni potentiated mineral deposition more effectively than early-phase exposure (Figure 3E). Mechanistically, qPCR analysis of osteogenic differentiation indicated that late-phase Ni did not significantly alter the early lineage marker *Runx2*, but significantly upregulated *Bglap*, a specific marker of late osteoblast maturation (Figure 3F).

### 2.4. Some Ni-Binding Proteins Can Be Enhanced upon Ni Stimulation During Osteogenic Mineralization

**Figure 4:**
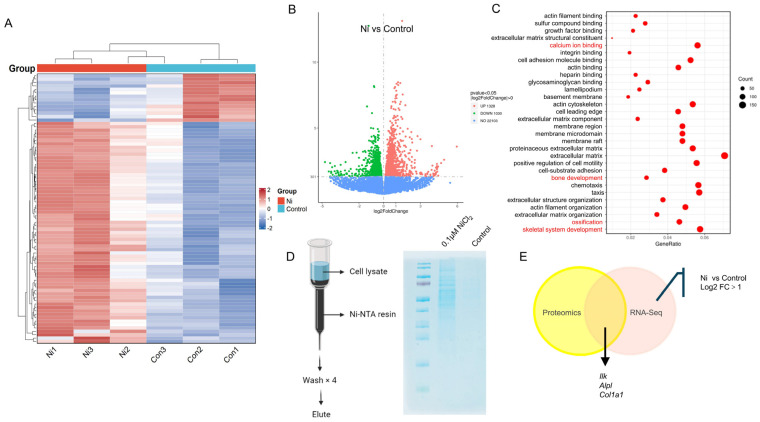
Integrated transcriptomic and Ni-affinity proteomic analyses identify Ni-responsive osteogenic programs and Ni-binding candidates during mineralization. (**A**) Heatmap of RNA-seq expression profiles from osteogenic cells mineralized for 7 days under control conditions or treated with 0.1 μM NiCl_2_, showing distinct clustering and broad differential expression between groups. (**B**) Volcano plot of differential gene expression between 0.1 μM NiCl_2_ and control groups; genes meeting |log_2_FC| > 1 are highlighted as significantly upregulated or downregulated. (**C**) Gene Ontology (GO) enrichment analysis of DEGs (log_2_FC > 1), demonstrating significant enrichment of osteogenesis- and bone development-related processes and structural remodeling pathways. The red indicates that nickel regulates osteogenesis-related signaling pathways. Dot size indicates gene count and dot color indicates adjusted *p* value (padj). (**D**) Workflow of Ni-NTA affinity pulldown using lysates from day-7 mineralizing osteogenic cells (±0.1 μM NiCl_2_) followed by LC–MS/MS. (**E**) Venn diagram showing the overlap between Ni-NTA-enriched proteomics candidates and RNA-seq upregulated genes (log_2_FC > 1, Ni vs. control).

RNA-seq was performed on BMSCs after 7 days of mineralization in the presence of 0.1 μM NiCl_2_ or under control conditions. The transcriptomic heatmap showed clear separation between NiCl_2_-treated and control samples with widespread differential expression, indicating a robust Ni-driven transcriptional reprogramming during osteogenic mineralization (Figure 4A). Consistently, the volcano plot highlighted a large set of differentially expressed genes (DEGs) meeting the threshold of |log_2_ fold change (FC)| > 1, including both significantly upregulated and downregulated transcripts in response to NiCl_2_ (Figure 4B). Gene Ontology enrichment analysis of these DEGs revealed significant overrepresentation of osteogenesis-related biological processes, such as bone development, ossification, and skeletal system development, together with pathways linked to matrix and structural remodeling, including extracellular matrix and extracellular structure organization (Figure 4C). To identify Ni-interacting proteins and determine whether Ni exposure alters the Ni-binding proteome, lysates from day-7 mineralizing cells (with or without 0.1 μM NiCl_2_ stimulation) were subjected to Ni-NTA affinity chromatography, which revealed an apparent increase in Ni-NTA-captured proteins in the NiCl_2_-treated group compared with controls, suggesting enhanced abundance of Ni-binding proteins upon Ni stimulation (Figure 4D). Finally, integrative multi-omics intersection of the Ni-NTA-enriched proteome with the RNA-seq upregulated genes (log_2_ FC > 1) identified the top three candidates that are both Ni-binding and Ni-inducible, prioritizing high-confidence targets for subsequent validation and mechanistic investigation (Figure 4E). Among these, *Ilk* exhibited the highest fold change, with a log_2_ FC of 1.3196.

### 2.5. Nickel Promotes Osteogenic Differentiation Through ILK-Dependent Activation of AKT–mTOR Signaling

**Figure 5:**
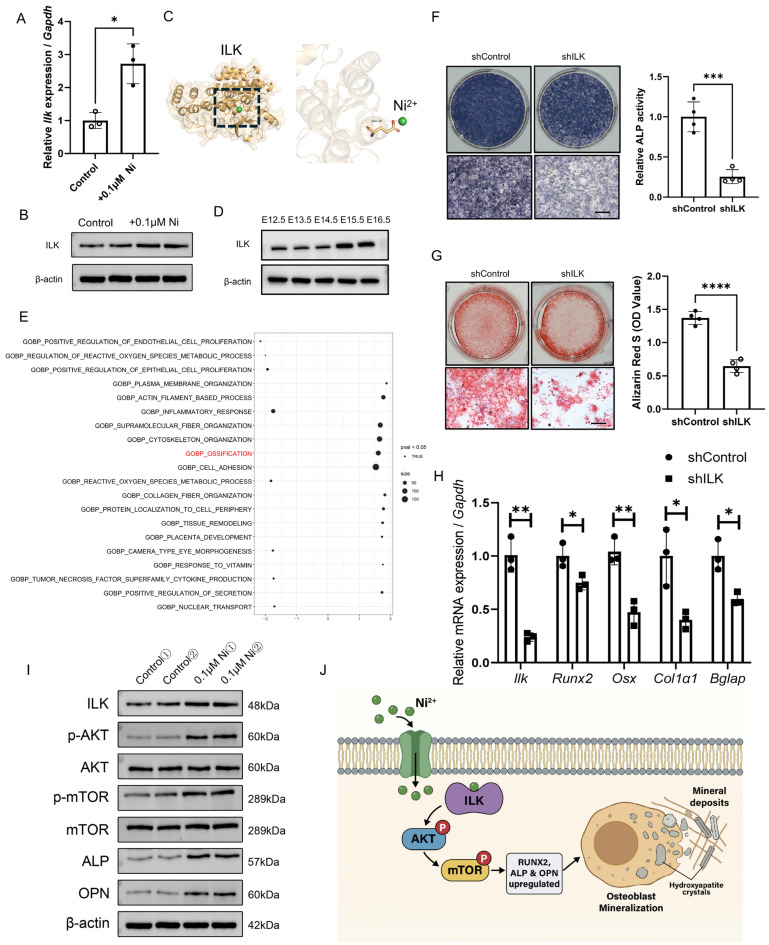
Nickel enhances osteogenic differentiation through ILK-dependent activation of AKT–mTOR signaling. (**A**,**B**) RT–qPCR (**A**) and immunoblot (**B**) analyses of ILK mRNA and protein in osteoblasts treated with 0.1 μM Ni or vehicle. (**C**) Molecular docking model of Ni^2+^ with ILK; the highlighted region marks the predicted Ni^2+^-binding pocket. (**D**) ILK expression in the calvaria across stages of skull development. (**E**) Pathway activity and gene-programme scores inferred from E17.5 single-cell RNA-seq data following in silico ILK knockout; osteogenesis-related programmes and bone formation-associated gene sets are shown. The red indicates the gene expression associated with the ossification pathway regulated by ILK. (**F**,**G**) Representative osteoblast differentiation assays following ILK knockdown, assessed by ALP staining and/or activity (**F**) and Alizarin Red S staining for mineral deposition (**G**). Scale bar = 200 μm. (**H**) RT–qPCR analysis of osteoblast differentiation-associated transcripts after ILK knockdown. (**I**) Immunoblot analysis of AKT and mTOR phosphorylation and indicated downstream proteins in osteoblasts treated with 0.1 μM Ni or vehicle. (**J**) Schematic model proposing that intracellular Ni^2+^ binds ILK, activates AKT–mTOR signaling, and promotes osteoblast differentiation and matrix mineralization. * *p* < 0.05, ** *p* < 0.01, *** *p* < 0.001, **** *p* < 0.0001.

Low-dose nickel (0.1 μM Ni) exposure robustly upregulated integrin-linked kinase (ILK) expression, as validated by qPCR and Western blotting experiments (Figure 5A,B). Molecular docking analyses uncovered a putative Ni^2+^-binding pocket within ILK (Figure 5C) with a calculated binding energy of −5.76 kcal/mol (Supplementary Table S1), demonstrating direct Ni^2+^–ILK physical interaction. ILK expression was dynamically elevated in the calvaria during skull morphogenesis (Figure 5D). Though scRNA-seq analysis of murine cranial tissue at E17.5 (GSE174716) demonstrated that *Ilk* gene was not specifically expressed in osteoblasts (Figure S5), pathway inference from E17.5 scRNA-seq revealed that simulated ILK ablation induced profound remodelling of osteogenesis-associated transcriptional programs and dysregulated bone formation-related gene expression (Figure 5E), establishing an essential role for ILK in osteogenic commitment and progression. Functionally, ILK knockdown markedly impaired osteoblast differentiation, as evidenced by reduced alkaline phosphatase (ALP) activity and alizarin red S staining (Figure 5F,G); qPCR experiments further confirmed perturbed expression of mineralization and bone differentiation-associated genes upon ILK silencing (Figure 5H and Figure S6). Mechanistically, 0.1 μM Ni stimulation triggered AKT and mTOR phosphorylation, concomitant with upregulated expression of downstream osteogenic-related proteins (Figure 5I). Collectively, these findings define a molecular mechanism whereby intracellular Ni^2+^ binds ILK to activate the AKT–mTOR signalling axis, thereby driving osteoblast differentiation and matrix mineral deposition (Figure 5J).

### 2.6. Ni-Pretreated Collagen Scaffolds Promote Calvarial Osteogenesis

To elucidate the structural basis of the interaction between nickel and collagen, we performed molecular docking simulations. Molecular docking analyses uncovered a putative Ni^2+^-binding pocket within COL1A1 with a calculated binding energy of −7.84 kcal/mol (Figure S7, Supplementary Table S1). The analysis identified specific high-affinity binding sites within the collagen triple helix, revealing that Ni^2+^ forms a stable coordination complex with Hydroxyproline-27 (Hyp27), Glycine-26 (Gly26) and Hyp27 (Figure S7). These findings provide structural evidence of a direct chemical coordination, corroborating our previous mass spectrometry data regarding the COL1A1-Ni^2+^ interaction. Leveraging this intrinsic affinity, we developed a collagen scaffold capable of retaining nickel ions for sustained delivery.

**Figure 6:**
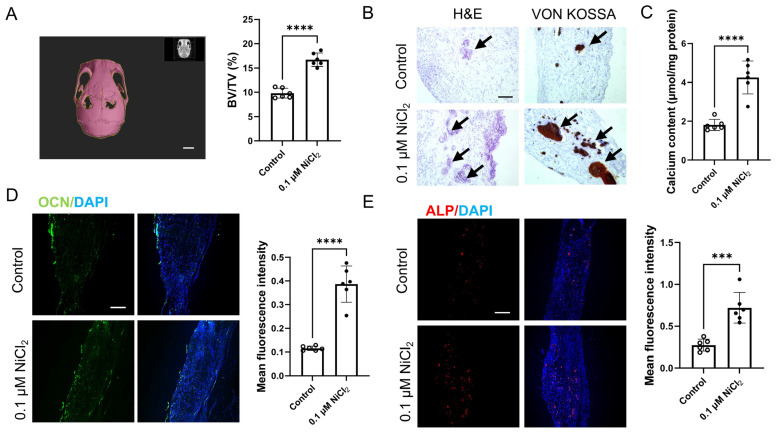
Bone regeneration in calvarial defect model at 8 weeks post-implantation of collagen scaffolds pre-treated with 0.1 μM Ni. (**A**) Quantification of BV/TV in the defect region between two groups by micro-CT analysis. (**B**) Representative H&E and Von Kossa staining of the defect area showing newly formed bone and mineralized matrix deposition. Black arrows indicate areas of mineral deposition. Scale bar = 100 μm. (**C**) Quantitative analysis of calcium content at the bone defect transplantation site between two groups. (**D**,**E**) Immunofluorescence staining showing ALP (red) and OCN (green) expression with DAPI (blue) nuclear counterstaining for comparison of osteogenic marker-positive cells between groups. Scale bar = 100 μm. *** *p* < 0.001, **** *p* < 0.0001.

To evaluate osteogenic efficacy in vivo, collagen scaffolds pre-treated with 0.1 μM Ni^2+^ were implanted into murine calvarial defects and analysed at 8 weeks post-implantation. Treatment with 0.1 µM Ni exhibited no systemic toxicity in major organs, including the liver, kidney and spleen (Figure S8). Micro-CT revealed a significant increase in BV/TV within defects treated with Ni-loaded scaffolds relative to controls, demonstrating enhanced osteogenesis (Figure 6A). H&E staining showed greater de novo bone formation in the Ni-treated group. Furthermore, Von Kossa staining and quantitative calcium assays revealed significantly augmented mineralized matrix deposition, indicating advanced maturation of the regenerated tissue in the presence of Ni (Figure 6B,C). At the cellular level, immunofluorescence staining demonstrated a marked abundance of cells positive for the early osteogenic marker OCN in the Ni-treated group (Figure 6D). In parallel, we observed an increase in ALP-positive cells, indicative of enhanced osteogenic matrix remodelling and bone formation (Figure 6E).

## 3. Discussion

Despite its expanding industrial use and well-documented toxicity at high doses [15,20,21], nickel is not currently recognized as an essential trace element in humans [4,9]. Our findings challenge this paradigm by revealing a dose-dependent role for nickel in skeletal development. Rather than acting merely as an environmental contaminant, nickel is dynamically coupled to the mineralizing microenvironment of the developing calvarium. Notably, nickel exerts a biphasic effect on both embryonic intramembranous ossification and adult bone microarchitecture: physiological concentrations promote bone formation, whereas elevated levels impair osteogenesis. These observations define a beneficial window for nickel during calvarial mineralization, beyond which it disrupts skeletal development.

To elucidate the pro-osteogenic mechanism of trace nickel, we focused on integrin signaling, a master mechanotransduction pathway in bone. ILK functions as a crucial downstream scaffold in this network, assembling the IPP complex (comprising ILK, PINCH, and parvin) to bridge the extracellular matrix with intracellular cascades, ultimately sustaining AKT phosphorylation [22,23]. Our molecular docking analyses revealed that Ni^2+^ stably coordinates within the N-terminal Glu-10 residue of ILK. As this residue is situated within the ankyrin repeat domain, which mediates PINCH binding [24], Ni^2+^ engagement may affect the local conformation of this region. Although this possibility has not been directly demonstrated for ILK, residue-specific Ni (II) binding has been reported to alter protein conformation in other systems, including Aβ peptides [25]. In addition, ATP binding has been reported to allosterically stabilize ILK [26], suggesting that the structural state of ILK can be modulated by molecular binding. Based on these observations, we postulate that Ni^2+^ engagement at Glu-10 may induce a local structural rearrangement that favors ILK interaction with PINCH, thereby stabilizing the IPP complex and ultimately contributing to enhanced downstream AKT activation. While our ICP-MS data conclusively map the spatial accumulation of Ni within the osseous matrix, the precise intracellular interactions remain to be visualized in vivo. Future investigations utilizing spatially resolved metallomics and targeted mutagenesis—specifically substituting the Glu-10 residue—are essential to definitively validate this ILK-centered mechanism and its direct contribution to ossification.

Following ILK activation, the AKT–mTOR pathway serves as the central intracellular node that transduces these signals into osteogenic action [27,28,29]. Previous studies have established that pharmacological or physiological stimulation of the PI3K–AKT–mTOR axis effectively enhances osteoblast function and mitigates bone loss in various osteoporosis models [30,31,32,33]. Conversely, supraphysiological doses of certain divalent cations, such as Sr^2+^, Mg^2+^, and Zn^2+^, have been shown to suppress this signaling axis [34,35,36]. Our findings align with these observations while introducing a novel paradigm: Ni^2+^, when restricted to its beneficial concentration, acts as a potent activator of the ILK–AKT–mTOR cascade, thus accelerating osteoblast maturation and bone development.

However, the beneficial effects of nickel are abruptly reversed at higher concentrations, primarily due to the disruption of cellular redox homeostasis. Toxic doses of nickel trigger a surge in ROS and suppress AKT phosphorylation, thus initiating autophagic and apoptotic programs [37,38,39]. In our study, the sustained transcriptional induction of Hmox1, a canonical cytoprotective enzyme mobilized during oxidative stress [40,41], indicates that cells exposed to high-dose Ni^2+^ mount a vigorous but ultimately insufficient antioxidant defense. The persistent activation of this pathway serves as a hallmark of unresolved intracellular redox disequilibrium [42]. Concurrently, elevated Nos2 expression drives the overproduction of inducible nitric oxide synthase (iNOS)-dependent nitric oxide (NO). The subsequent reaction between excess NO and superoxide generates peroxynitrite (ONOO^−^), a highly destructive oxidant that perpetuates lipid peroxidation, protein nitration, and DNA damage [43]. This severe cytotoxic stress cascade explains the impaired bone formation observed at high doses.

The biphasic nature of nickel identified herein bears profound translational implications, particularly for the design of orthopedic and dental biomaterials. Nickel is heavily utilized in implant alloys, such as Nitinol and stainless steels, yet the biological ramifications of its gradual release into the peri-implant environment remain understudied. Consistent with a prior report by Vyas et al., which demonstrated that low-level NiO doping in bioglass matrices accelerated cell proliferation [44], our in vivo experiments confirmed that collagen membranes doped with an optimized concentration of nickel significantly accelerated calvarial bone regeneration. However, uncontrolled nickel leaching from traditional implants poses severe clinical risks, notably triggering excessive inflammation, chronic oxidative stress, and tissue degradation, especially in patients with autoimmune conditions or metal hypersensitivity [12,14]. By mapping the precise “beneficial window” for nickel, our study highlights the urgent necessity for next-generation biomaterials equipped with tunable release kinetics. Engineering implants to maintain local nickel exposure strictly within this pro-osteogenic range could maximize osseointegration and accelerate healing, while systematically avoiding the pitfalls of local cytotoxicity and implant failure.

Finally, while our data establish the physiological accumulation of nickel within bone and its impact on microarchitectural remodeling, certain limitations warrant consideration. First, strict control over the administered nickel dose is imperative, yet our current study lacks systemic serum nickel quantification. Second, the cross-talk or competition between nickel and other essential trace metals (such as calcium or zinc) remains unexplored. Comprehensive pharmacokinetic studies are therefore urgently needed to map the systemic biodistribution, compartmental partitioning, and long-term clearance kinetics of nickel following localized administration in bone defects.

## 4. Method and Materials

### 4.1. Mice

All mice experiments were approved by the Animal Care and Ethics Committee of Sun Yat-sen University (the approval number of the laboratory: 2025003883). For the nickel-free group, offspring were maintained from birth until 8 weeks of age on a nickel-free diet (purchased from Trophic Animal Feed High-tech Co., Ltd., Nantong, Jiangsu, China) that strictly matched the standard control diet in all essential trace elements, except for the omission of nickel. For the late stage of high-nickel intake group, 5-week-old male mice received drinking water respectively supplemented with 2.5 mmol/L NiCl_2_ and 7.5 mmol/L NiCl_2_ for the subsequent 3 weeks. For the early stage of high-nickel intake group, 2-week-old male mice received drinking water supplemented with 7.5 mmol/L NiCl_2_ for the subsequent 6 weeks.

To evaluate dynamic bone formation and mineralization, sequential fluorescent labeling was performed. Seven days before euthanasia, each mouse was intraperitoneally injected with 100 μL of calcein (5 mg/mL, pH 7.2–7.4). Five days later (i.e., 2 days before euthanasia), mice received an intraperitoneal injection of 100 μL of Alizarin Red S (3 mg/mL, pH 7.2–7.4).

At the end of the experimental period, femurs were harvested. Undecalcified bone samples were embedded, and longitudinal cryostat sections (10 μm thickness) were prepared. Sections were imaged under a fluorescence microscope to visualize the green (calcein) and red (Alizarin Red S) fluorescent labels, allowing quantification of bone formation rate.

### 4.2. Immunofluorescence Staining

For tissue sections, samples were fixed overnight in 4% PFA at RT. Antigen retrieval was achieved by heating slides in citrate buffer (pH 6.0) at 95 °C for 20 min. Sections were subsequently permeabilized with 0.1% Triton X-100 in PBS for 10 min and blocked with 5% normal serum (species-matched to secondary antibodies) plus 1% BSA for 1 h at RT. Primary antibodies diluted in 1% BSA/0.1% Tween-20/PBS were applied overnight at 4 °C. Following three washes in PBST, Alexa Fluor-conjugated secondary antibodies (1:500; Invitrogen, Carlsbad, CA, USA) were incubated for 1 h at RT protected from light. Nuclei were stained with DAPI (1 μg/mL) for 5 min.

Imaging was performed on a Leica TCS SP8 confocal microscope with ×63 oil immersion objectives (Leica Microsystems GmbH, Wetzlar, Hesse, Germany). Z-stack images (step size 0.5 μm) were acquired and processed using Leica LAS X software (version 3.7). Fluorescence intensity and colocalization analyses were conducted using ImageJ/Fiji (version 2.3.0).

The primary antibodies used in this study are listed below:

anti-RUNX2 (ab236639, Abcam, Cambridge, MA, USA), anti-ALP (ET1601-21, HUABIO, Hangzhou, Zhejiang, China), anti-OPN antibody (ab283656, Abcam, USA), anti-Heme Oxygenase 1 antibody (ab68477, Abcam, USA), Anti-Collagen I antibody (ab138492, Abcam, USA) and anti-iNOS antibody (ab178945, Abcam, USA).

### 4.3. Intracellular ROS Detection

BMSCs (untreated or NiCl_2_-stimulated for 12, 24 or 48 h) were prepared for intracellular ROS detection. Cells gently rinsed twice with 37 °C pre-warmed sterile PBS (1 min static incubation post each rinse, followed by aspiration); 1 mL of 10 μmol/L DCFH-DA working solution was then added per well, and the plate was swirled for uniform cell coverage, wrapped in aluminum foil for light protection and incubated for 30 min in a humidified 37 °C, 5% CO_2_ incubator; residual DCFH-DA was subsequently aspirated, cells rinsed three times with pre-warmed PBS to remove unincorporated probe, and fluorescence intensity visualized via fluorescence microscope, with higher intensity indicating greater intracellular ROS accumulation.

### 4.4. Micro-Computed Tomography (Micro-CT)

Femurs were imaged using a SCANCO Medical μCT 50 system (Brüttisellen, Switzerland). Scans were acquired at an isotropic voxel resolution of 6 μm with an X-ray tube voltage of 55 kV, generating sequential tomographic slices. Three-dimensional reconstructions were created using CTAn (version 1.23.0.2+) and CTVox (version 3.3.0 r1383) software. For trabecular bone analysis, the region of interest (ROI) was defined in the distal femoral metaphysis (excluding cortical bone). For cortical bone, the ROI was set at the mid-diaphysis. Key structural parameters were quantified, including bone volume fraction (BV/TV), trabecular thickness (Tb.Th), trabecular number (Tb.N), trabecular separation (Tb.Sp), cortical thickness (Ct.Th), cortical bone area (Ct.B.Ar), and total cortical area (Ct.T.Ar).

### 4.5. Histological Analysis

Cranium and femur samples were decalcified using a commercial ethylenediaminetetraacetic acid (EDTA)-based solution (Servicebio Technology Co., Ltd., Wuhan, China) at room temperature for 3 months. Following decalcification, tissues underwent graded ethanol dehydration, were embedded in paraffin, and sectioned at a thickness of 5 μm. Hematoxylin and eosin (H&E) staining, along with von Kossa staining for mineralized bone, was carried out following the manufacturer’s protocols (Solarbio, Beijing, China).

### 4.6. In Vitro Osteogenic Differentiation

Bone marrow mesenchymal stem cells (BMSCs) and calvarial suture-derived stem cells were maintained in osteogenic induction medium supplemented with 10 mM β-glycerophosphate, 50 μg/mL ascorbic acid (vitamin C), and 1 nM dexamethasone. Osteogenic differentiation was induced for periods of 7 and 14 days.

Following 7 and 14 days of osteogenic culture, alkaline phosphatase (ALP) staining was conducted using a BCIP/NBT Chromogenic Kit (Beyotime Biotechnology, Shanghai, China), and ALP activity was quantified with an ALP Detection Kit (Beyotime Biotechnology, Shanghai, China), both according to the manufacturer’s guidelines. Gene and protein expression profiles were assessed by quantitative real-time reverse transcription polymerase chain reaction (qRT-PCR) and Western blotting, respectively. The mRNA levels of key osteogenic markers including *Osx*, *Runx2*, *Col1a1*, and *Bglap* were measured via qRT-PCR.

### 4.7. RT-qPCR

Total RNA was isolated from cells using TRIzol Reagent (Invitrogen, Carlsbad, CA, USA) as per the supplier’s protocol. RNA quantity and purity were evaluated on a NanoDrop 2000 spectrophotometer (Thermo Fisher Scientific, Waltham, MA, USA). Complementary DNA (cDNA) was reverse-transcribed with the PrimeScript™ RT Reagent Kit (Takara Bio Inc., Kusatsu, Shiga, Japan). qRT-PCR reactions were set up using ChamQ SYBR qPCR Master Mix (Vazyme, Nanjing, China), and relative gene expression was calculated by the 2^−ΔΔCt^ method, normalized to glyceraldehyde-3-phosphate dehydrogenase (*Gapdh*) as the housekeeping gene. All assays were conducted in triplicate. Primer sequences are provided in Table 1.

### 4.8. Western Blot Analysis

Cells were lysed in radioimmunoprecipitation assay (RIPA) buffer (Sigma-Aldrich, St. Louis, MO, USA) containing a cocktail of protease and phosphatase inhibitors (Roche, Basel, Switzerland). Protein concentration in the lysates was measured using the BCA Protein Assay Kit (Beyotime Biotechnology, Shanghai, China). Equal protein quantities were resolved by sodium dodecyl sulfate–polyacrylamide gel electrophoresis (SDS-PAGE) and electrotransferred onto polyvinylidene difluoride (PVDF) membranes. Membranes were blocked in 5% non-fat dry milk for 2 h at room temperature, followed by overnight incubation with primary antibodies at 4 °C. Subsequently, membranes were incubated with horseradish peroxidase (HRP)-linked secondary antibodies for 1 h at room temperature. Immunoreactive bands were detected with an enhanced chemiluminescence (ECL) reagent (Millipore, Billerica, MA, USA). Densitometric analysis of band intensities was performed using ImageJ software. All experiments were run in triplicate.

The primary antibodies used in this study are listed below:

anti-β-ACTIN (AC026, ABclonal, Wuhan, Hubei, China), anti-RUNX2 (ab236639, Abcam, Cambridge, MA, USA), anti-ILK (ab76468, Abcam, Cambridge, MA, USA), anti-mTOR (phospho S2448) antibody (ab109268, Abcam, Cambridge, MA, USA), anti-AKT (phospho T308) antibody (ab38449, Abcam, Cambridge, MA, USA), AKT Pan Polyclonal Antibody (44-609G, Thermo Fisher Scientific, Waltham, MA, USA), mTOR Polyclonal Antibody (PA5-34663, Thermo Fisher Scientific, Waltham, MA, USA), anti-OPN antibody (ab283656, Abcam, Cambridge, MA, USA) and anti-ALP (ab229126, Abcam, Cambridge, MA, USA).

### 4.9. Lentiviral-Mediated ILK Knockdown in BMSCs

BMSCs were seeded in 6-well plates and cultured to 30–40% confluency under standard conditions (37 °C, 5% CO_2_). sh-ILK lentivirus (sc-35667-V, Santa Cruz Biotechnology, Dallas, TX, USA) or scramble shRNA lentivirus (negative control) was diluted in serum-free α-MEM, mixed with polybrene (5 μg/mL) to enhance transduction efficiency, then added to each well. After 8 h of incubation, the lentivirus-containing medium was replaced with complete α-MEM (10% FBS, 1% penicillin–streptomycin), and cells were cultured for an additional 48 h. To obtain stably transfected cell lines, puromycin (2 μg/mL) was added to the medium for 7 days of selection, with medium renewal every 48 h; ILK knockdown efficiency was subsequently verified by Western blot and qRT-PCR to confirm successful establishment of ILK-silenced BMSCs.

### 4.10. Molecular Docking Analysis

Semi-flexible docking was employed to generate stable ligand–receptor complexes. Molecular preparations were performed using AutoDock 1.5.6 software. For ions, the AutoDock preparation protocol was applied. Docking grid parameters were subsequently defined in the Grid module. The grid box was set to 126 × 126 × 126 Å to encompass the entire protein. The grid spacing was fixed at 0.60 Å, and the grid maps were calculated and saved accordingly. All other parameters were retained at their default values. The resulting docking poses were visualized to allow direct observation of the ligand–receptor binding modes and to facilitate further analysis of complex stability and key intermolecular interactions.

### 4.11. In Vivo Bone Defect Model

Under anesthesia, the skull was surgically exposed, and bilateral standardized critical-size calvarial defects (3 mm diameter) were created in each mouse using a saline-irrigated dental trephine. Ni-functionalized scaffolds were prepared by immersing Type I collagen-based membranes in 0.1 μM NiCl_2_ solution at 37 °C for 2 h prior to implantation. In the control group, defects were left untreated or filled only with plain Type I collagen-based membrane, while experimental groups received full coverage with the Ni-functionalized scaffolds. Wounds were closed by careful suturing, and animals were administered penicillin for three consecutive days postoperatively. At 8 weeks post-surgery, mice were euthanized by anesthetic overdose, and cranial specimens were harvested and fixed in 4% paraformaldehyde.

### 4.12. Trace Element Quantification

Serum samples were digested in concentrated HNO_3_ and H_2_O_2_ using a microwave digestion system. The total Ni concentration was subsequently quantified using Inductively Coupled Plasma Mass Spectrometry (ICP-MS, Agilent 7800, Santa Clara, CA, USA), utilizing standard calibration curves and internal standards to ensure accuracy. Bone tissues were mounted on a stable support carrier, and in situ laser ablation inductively coupled plasma mass spectrometry (LA-ICP-MS) was directly performed to determine the metal contents in bone.

### 4.13. Statistical Analysis

Data are expressed as mean ± standard deviation (SD). Statistical comparisons among three or more groups were performed by one-way analysis of variance (ANOVA) followed by Tukey’s post hoc test. Differences between two groups were evaluated using the unpaired Student’s *t*-test. All statistical analyses were conducted with GraphPad Prism software (version 10.1.2).

## 5. Conclusions

In summary, this study fundamentally redefines the biological identity of Ni in skeletal biology, establishing it as a “double-edged sword.” We demonstrate that Ni naturally accumulates in the developing cranium and exhibits a robust positive correlation with progressive matrix mineralization. Functionally, Ni operates under a stringent biphasic, dose-dependent paradigm: while Ni deprivation precipitates growth retardation and excessive Ni exposure triggers ROS-mediated osteotoxicity, low-level physiological Ni serves as a potent pro-osteogenic signal. Mechanistically, trace Ni^2+^ engages ILK, thereby activating the AKT–mTOR signaling cascade to drive osteoblast maturation. Furthermore, the efficacious deployment of Ni-loaded collagen scaffolds for calvarial defect repair underscores the tremendous translational promise of harnessing trace-metal interactions for regenerative therapeutics. Ultimately, this work shifts the perspective of nickel from a purely toxic environmental contaminant to a dose-sensitive, stage-dependent regulator of bone homeostasis.

## Figures and Tables

**Table 1:** Primers used for RT-qPCR analysis.

Gene	Forward Primer	Reverse Primer
*Col1a1*	GCTCCTCTTAGGGGCCACT	CCACGTCTCACCATTGGGG
*Runx2*	GACTGTGGTTACCGTCATGGC	ACTTGGTTTTTCATAACAGCGGA
*Osx*	ATGGCGTCCTCTCTGCTTG	TGAAAGGTCAGCGTATGGCTT
*Bglap*	GCAATAAGGTAGTGAACAGACTCC	GCGTTTGTAGGCGGTCTTCAAG
*Ilk*	GTGCTGAAGGTTCGTGACTGGA	TCCAGTGTGTGATGAGGGTTGG
*Gapdh*	AGGTCGGTGTGAACGGATTTG	TGTAGACCATGTAGTTGAGGTCA

## Data Availability

The data presented in this study are openly available in GEO at https://www.ncbi.nlm.nih.gov/geo/query/acc.cgi?acc=GSE322756, last update date 3 March 2026, reference number elktkwielbcxtqz.

## Supplementary Materials

The following supporting information can be downloaded at: https://www.mdpi.com/article/10.3390/ijms27104538/s1.
