# Supplementary material for: Dose-Dependent Effects of Nickel on Skeletal Development: Physiological Necessity and the Threshold of Toxicity
Source: Int J Mol Sci. 2026 May 18;27(10):4538. doi: 10.3390/ijms27104538 (PMC13207421; doi:10.3390/ijms27104538)
Supplement: Supplementary file 1 [file ijms-27-04538-s001.zip › ijms-4256077-supplementary.pdf]

Dose-Dependent Effects of Nickel on Skeletal Development: Physiological Necessity and the Threshold of Toxicity

This document includes: Supplementary Table S1 and Supplementary Figure S1 to Supplementary Figure S8.

Supplementary Table S1. Molecular docking binding energies of potential binding pockets of Ni^{2+}-ILK and Ni^{2+}-COL1A1.

	Binding Energy (kcal/mol)
Ni^{2+} and ILK	-5.76
Ni^{2+} and COL1A1	-7.84

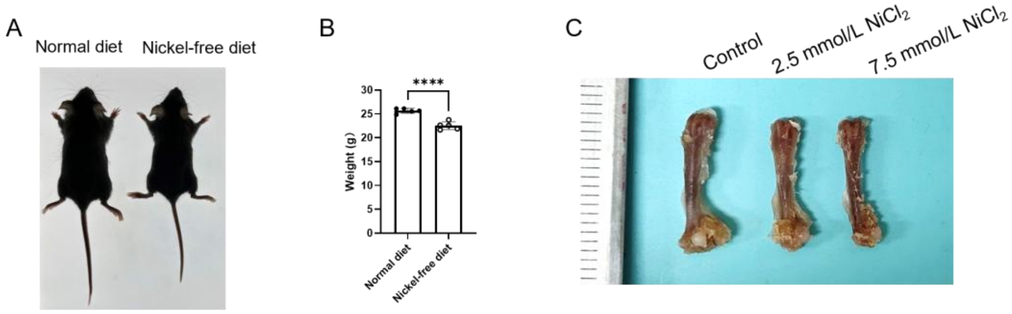

Figure S1. Nickel availability modulates body size and long-bone growth in mice. **A** Representative images showing overall body habitus of mice fed a nickel-free diet compared with control diet, indicating a smaller body size in the nickel-free group. **B** Body weight comparison between control and nickel-free diet groups. **C** Representative femora from mice in the control, 2.5 mmol/L $NiCl_2$ and 7.5 mmol/L $NiCl_2$ groups; femora from the 7.5 mmol/L $NiCl_2$ group appear smaller, consistent with impaired long-bone growth at higher nickel exposure.

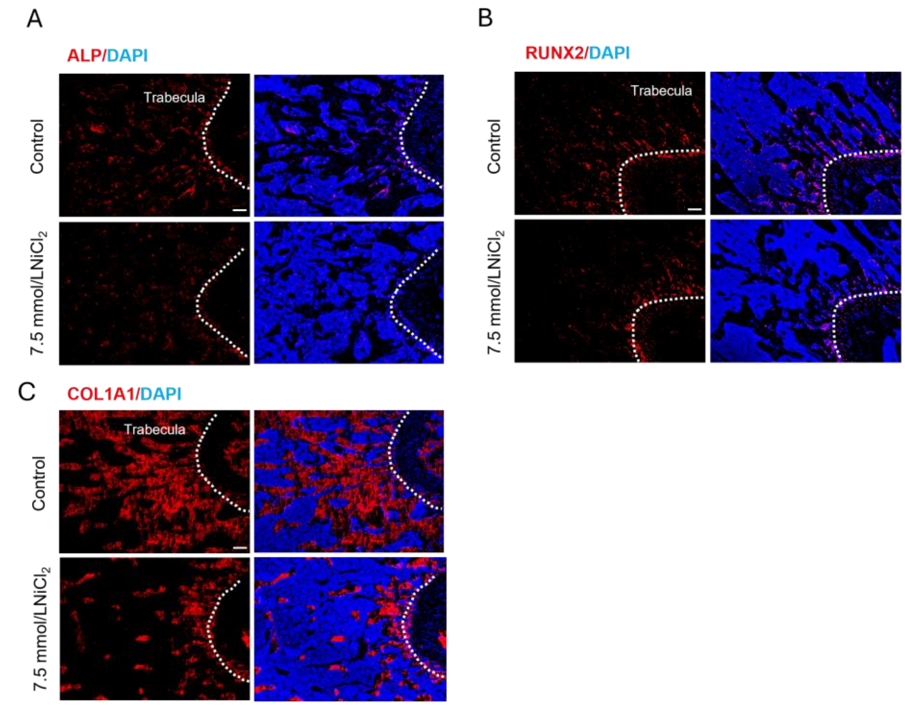

Figure S2. High nickel exposure reduces RUNX2, alkaline phosphatase (ALP) and COL1A1 expression in the mouse femur. **A** Representative immunofluorescence images of ALP (red) in femoral sections from control mice and mice exposed to 7.5 mmol/L Ni in the early-stage of nickel intake. **B** Representative immunofluorescence images of RUNX2 (red) in femoral sections from control mice and mice exposed to 7.5 mmol/L Ni in the early-stage of nickel intake. **C** Representative immunofluorescence images of COL1A1 (red) in femoral sections from control mice and mice exposed to 7.5 mmol/L Ni in the early-stage of nickel intake.Nuclei were counterstained with DAPI (blue). Scale bar = 100 µm.

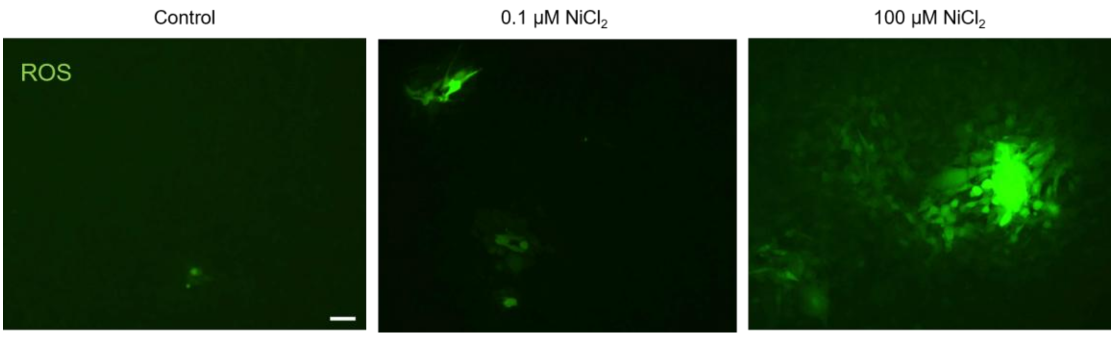

Figure S3. High-dose $NiCl_2$ elevates intracellular ROS in BMSCs. Representative fluorescence micrographs of ROS staining in BMSCs following 24 h exposure to control, 0.1 μM $NiCl_2$ (low-dose nickel), or 100 μM $NiCl_2$ (high-dose nickel). Scale bars=100μm

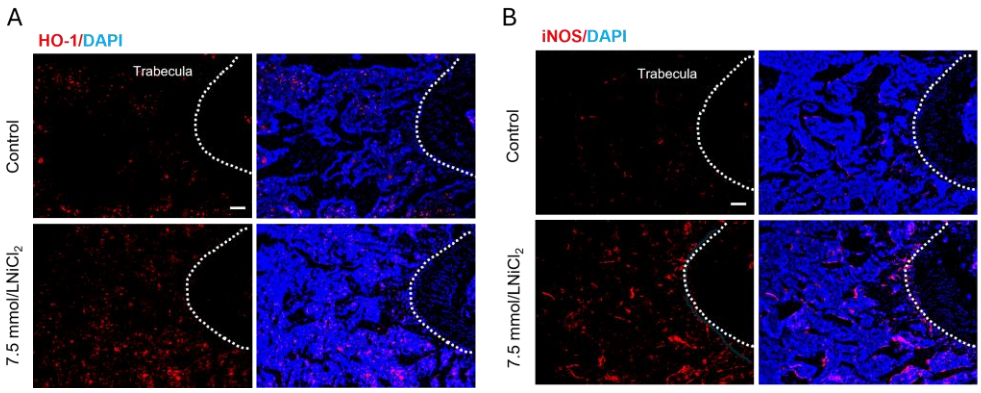

Figure S4. High nickel exposure induces HO-1 and iNOS expression in the mouse femur. **A** Representative immunofluorescence images of HO-1(red) in femoral sections from control mice and mice exposed to 7.5 mmol/L Ni in the early-stage of nickel intake. **B** Representative immunofluorescence images of iNOS (red) in femoral sections from control mice and mice exposed to 7.5 mmol/L Ni in the early-stage of nickel intake. Nuclei were counterstained with DAPI (blue). Scale bar = 100 μm

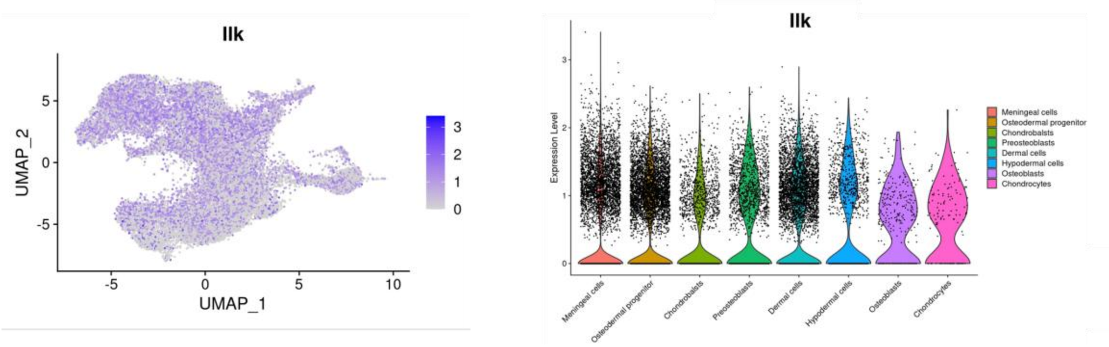

Figure S5. *Ilk* is broadly expressed across cranial cell populations in E17.5 mouse scRNA-seq (GSE174716).

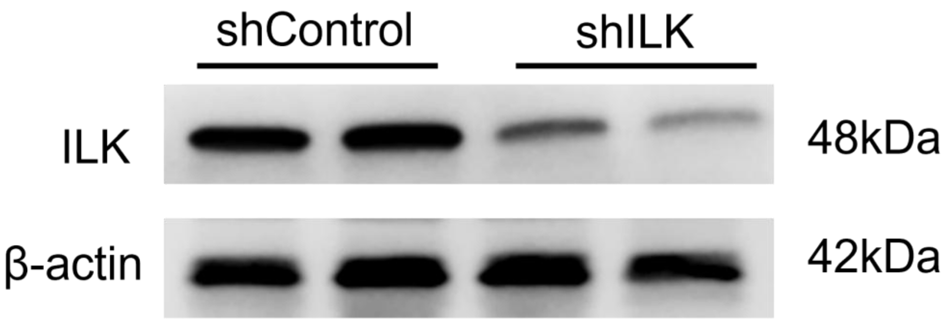

Figure S6. Western blot analysis showing ILK protein expression in shControl and shILK groups. β-actin was used as the control.

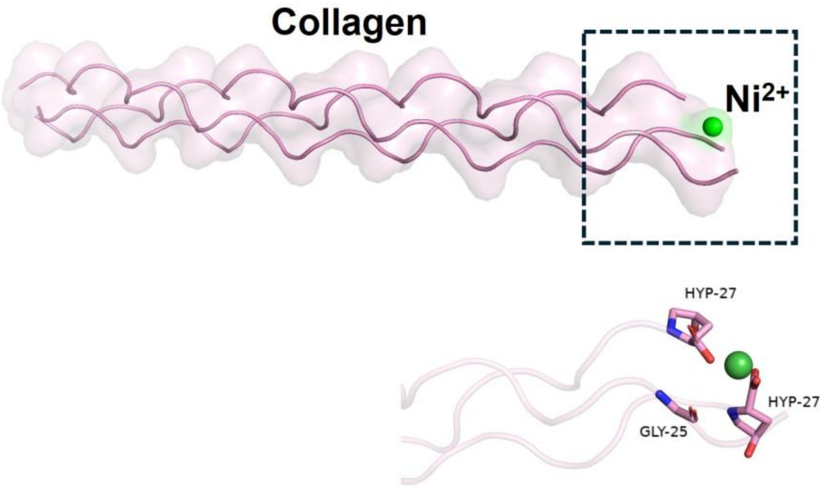

Figure S7. Molecular docking simulation analysis of the binding sites between Ni^{2+} and collagen.

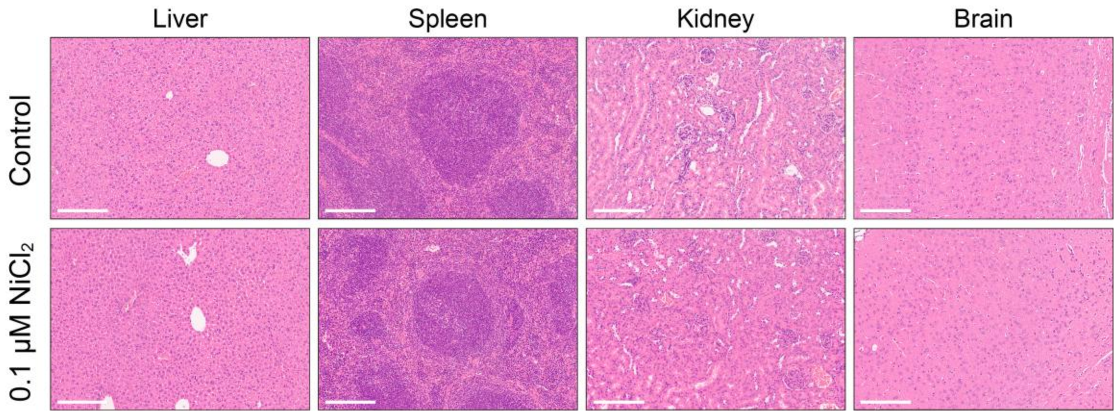

Figure S8. Representative H&E staining of the liver, spleen, kidney, and brain from control and 0.1 μM $NiCl_2$-treated mice. Scale bar = 200 μm.
